# Compartmentalized thymidine phosphorylation by mitochondrial nucleotide kinases TK2 and CMPK2

**DOI:** 10.1016/j.jbc.2025.110733

**Published:** 2025-09-16

**Authors:** Avery S. Ward, Vasudeva G. Kamath, Chia-Heng Hsiung, Zachary J. Lizenby, Alexander G. Gillish, D. Stave Kohtz, Edward E. McKee

**Affiliations:** 1 Department of Foundational Sciences, College of Medicine, Central Michigan University, Mount Pleasant, Michigan, USA; 2 Basic Medical Sciences, University of Arizona College of Medicine, Phoenix, Arizona, USA; 3 Westlake Laboratory of Life Sciences and Biomedicine, Hangzhou, Zhejiang, China

**Keywords:** thymidine kinase 2, mitochondrial metabolism, mitochondrial disease, nucleoside–nucleotide biosynthesis, nucleoside–nucleotide metabolism, cytidine monophosphate kinase 2

## Abstract

Deoxynucleotides (dNTPs) in postmitotic tissues rely on deoxynucleoside salvage pathways in order to repair and replicate nuclear and mitochondrial DNA. Previous work from our laboratory showed in perfused rat hearts and isolated mitochondria that the only substrate for thymidine triphosphate synthesis is thymidine. When thymidylate (thymidine monophosphate [TMP]) is provided to bypass thymidine kinase 2 (TK2), the substrate is readily dephosphorylated to thymidine before salvage occurs, suggesting compartmentalization within the heart mitochondrial matrix. The goal of this work extends these findings in the heart to mitochondria from other postmitotic tissues, including rat liver, kidney, and brain. Using azidothymidine to block mitochondrial TK2, we demonstrate that TMP cannot serve as a precursor for thymidine triphosphate synthesis in isolated mitochondria from any of these tissues unless it is dephosphorylated to thymidine first. Broken mitochondria incubated with labeled TMP showed similar results as intact mitochondria, suggesting the findings are not related to TMP transport across the inner mitochondrial membrane. Further, using proximity labeling with immunofluorescence microscopy, we provide evidence supporting the hypothesis that TMP compartmentation is accounted for by the interaction of TK2 and cytidine/uridine monophosphate kinase 2 (CMPK2) in the mitochondria. Differential fraction experiments provide additional evidence that association with TK2 allows CMPK2 to display cytosolic thymidylate kinase 2 activity. Together, the results indicate that a two-step phosphorylation of thymidine to TDP occurs because the proximity of TK2 and CMPK2 in the mitochondria prevents TMP from diffusing from the two enzymes.

Replicating cells have a high demand for deoxynucleoside triphosphates (dNTPs). As a result, the dNTP pool sizes and the enzymes that synthesize dNTPs are typically well expressed (1) and may include *de novo* synthesis as well as synthesis from the salvage pathway. In postmitotic tissues, dNTPs are only required for nuclear DNA repair and mitochondrial DNA replication, and the dNTP pool sizes are much reduced (2, 3). As such, the enzymes of dNTP synthesis are thought to be poorly expressed, and the salvage pathways predominate. Thymidine triphosphate (TTP) is the only dNTP that is not a direct product of ribonucleotide reductase and has no ribonucleotide equivalent. In the S-phase of mitotic tissues, the level of TTP is high, and TTP may be synthesized from *de novo* dUMP by thymidylate synthase and methylenetetrahydrofolate or from *de novo* dCMP following deamination to dUMP (4). An additional major route of synthesis of TTP in S-phase is from the salvage of thymidine by cytosolic thymidine kinase 1 (TK1) or, to a much lesser extent, mitochondrial thymidine kinase 2 (TK2) (Fig. 1), which is constitutively expressed at a much lower level than TK1. Thymidine monophosphate (TMP) synthesized in the cytosol is further phosphorylated by a cytosolic thymidylate kinase (TMPK) and cytosolic nucleoside diphosphate kinase to TTP. During S-phase, the majority of TTP used for mitochondrial DNA replication is transported from the cytosolic TTP salvage and *de novo* pathway (5). In postmitotic tissues, TK1 is inactive, and in the heart, the enzymes of *de novo* TTP synthesis are inactive (6). In these tissues, TTP synthesis to support mitochondrial 10.13039/100026054DNA replication is supplied by mitochondrial TK2 and the mitochondrial phosphorylation pathway (Fig. 1) (6). There is significant evidence that the cytoplasmic and mitochondrial deoxynucleoside and deoxynucleotide phosphate pools communicate by specific inner membrane transporters (7, 8, 9, 10).

**Figure 1:**
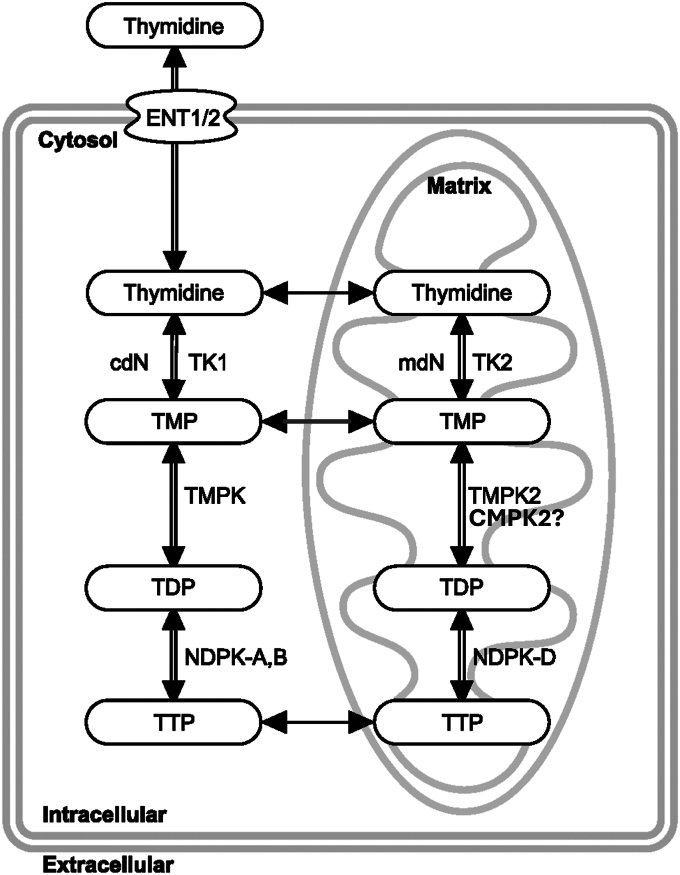
**Model of thymidine triphosphate (TTP) salvage pathway in cytosol and mitochondrial matrix (see text for details)**. cdN, cytosolic deoxynucleotidase; ENT1/2, equilibrative nucleoside transporter 1/2; mdN, mitochondrial deoxynucleotidase; NDPK-A, B, cytosolic nucleoside diphosphate kinase; NDPK-D, mitochondrial nucleoside diphosphate kinase; TK1/2, thymidine kinase 1/2; TMPK, cytosolic thymidylate kinase; TMPK2, presumptive mitochondrial thymidylate kinase (CMPK2).

The phosphorylation of TK2-synthesized TMP in mitochondria requires an as-yet-unidentified TMP kinase. An enzyme identified as a mitochondrial TMPK2 by sequence (11) and by showing increased TTP levels in HeLa cells when overexpressed is now known as cytidine/uridine monophosphate kinase 2 (CMPK2) (12), which in purified form *in vitro* phosphorylates dUMP and dCMP, and to a lesser extent UMP and CMP, to diphosphates, but not TMP (11, 12). The last phosphorylation of deoxynucleotide diphosphates is mediated by a mitochondrial matrix nucleoside diphosphokinase, which appears to require activity of succinyl-CoA transferase (an ADP-dependent and a GDP-dependent form) and γ-aminobutyric acid transaminase (13). Deficiencies of any of the enzymes in the mitochondrial deoxynucleoside phosphorylation pathway have been reported to lead to mitochondrial DNA depletion disease. Humans known to have mutations in *TK2* suffer from a severe myopathy that is characterized by mitochondrial DNA depletion syndrome and is typically lethal in the first few years of life (14). It was shown that low levels of TTP were associated with this disorder (15). A potential treatment for this disorder was the provision of supplemental dCMP and TMP, which if it crossed the plasma and inner mitochondrial membrane, would bypass the defective enzyme (16).

In earlier work, we demonstrated in adult isolated rat heart perfusion that the synthesis of TTP depends solely on the salvage of thymidine by TK2 (6). Additional work in isolated heart mitochondria demonstrated that a bolus of unlabeled TMP failed to inhibit the conversion of 3H-thymidine to 3H-TTP (17), suggesting compartmentalization of TMP pools as the added unlabeled TMP apparently did not mix and dilute the 3H-TMP arising from 3H-thymidine. Last, we have shown in heart mitochondria that 3H-thymidine was not only a better precursor for 3H-TTP synthesis than 3H-TMP but also that synthesis of 3H-TTP from 3H-TMP required the dephosphorylation of 3H-TMP to 3H-thymidine prior to the synthesis of 3H-TTP (18). If these results are typical in other tissues, then we would not expect that supplemental dCMP and TMP would correct TK2 deficiency even if they crossed the plasma membrane. It has since been shown that the effects of dCMP and TMP supplementation in both the mouse model and in compassionate human therapy in TK2-deficient patients could be recapitulated with supplementation with deoxycytidine and thymidine (19, 20, 21). The treatment led to significant improvement in the mouse model and in the human patients in both function and lifespan, although, they were still left with considerable disease (19). The physiological concentration of deoxycytidine and thymidine is thought to be in the 1 μM range (22). The treatment dose for these studies was typically in the 400 to 520 mg/kg range, and while blood levels were not provided, this would place the deoxynucleosides in the millimolar range, about 1000 times higher than physiological. While the mechanism of the noted improvement is largely unknown, it may be related to an increase in the phosphorylation of the very high concentrations of deoxynucleosides by perhaps residual TK1 or perhaps low phosphorylation activity of other active deoxynucleoside kinases, such as dCK.

The first goal of the current investigation was to determine if the observation that TMP must be dephosphorylated to thymidine before it can serve as a substrate for TTP synthesis in heart mitochondria was unique to the heart or was a more general property of rodent mitochondria in adult postmitotic tissues. To accomplish this, mitochondria were isolated from liver, kidney, and brain, and the conversion of 3H-thymidine and 3H-TMP to 3H-TTP was examined. Results will show that the synthesis of 3H-TTP from 3H-thymidine and 3H-TMP is more robust in intact and broken mitochondria from liver, kidney, and brain than was observed in intact and broken heart mitochondria, but that all tissues required the dephosphorylation of TMP prior to TTP synthesis, demonstrating compartmentalization of TMP not accounted for by the mitochondrial membranes. The second goal of this work was to begin to understand the mechanism of TMP compartmentalization. As noted above, CMPK2 was originally identified by sequence as a TMPK, and it was shown that overexpression of this enzyme led to increased steady-state levels of TTP. However, in purified form, it phosphorylates dUMP and dCMP, and to a lesser extent, UMP and CMP, but not TMP (11, 12). We show with proximity labeling and immunofluorescence analysis that CMPK2 and TK2 associate with each other and hypothesize that when CMPK2 associates with TK2, it acquires TMPK activity and converts enzyme-bound TMP to TDP, preventing the diffusion of TMP from the enzyme, accounting for TMP compartmentalization.

## Results

### Metabolism of 3H-thymidine and 3H-TMP in isolated rat tissue mitochondria

**Figure 2:**
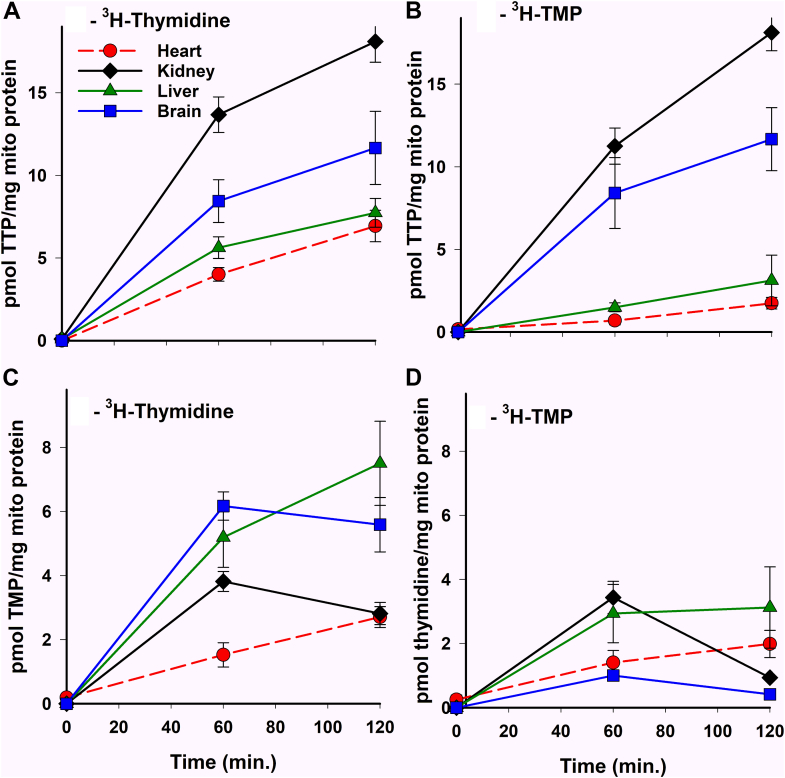
**3H-thymidine and 3H-TMP metabolism in isolated intact tissue mitochondria**. Freshly isolated intact liver, brain, and kidney mitochondria (4 mg protein/ml) were incubated with 100 nM 3H-thymidine or 3H-TMP (∼2200 dpm/pmol), respectively, in incubation medium (18) at 30 °C for 0, 60, and 120 min, and processed as described in the *Experimental procedures* section. The 3H-products were identified and quantitated by UPLC as described in the *Experimental procedures* section. Results were expressed as pmol product/mg mitochondrial protein and plotted against time. Previous data from identical experiments in intact heart mitochondria were plotted along with tissue mitochondria results for comparison. *A*, an amount of 3H-TTP was observed when exogenous 3H-thymidine was provided. *B*, an amount of 3H-TTP was observed when exogenous 3H-TMP was provided. *C*, an amount of 3H-TMP was observed when exogenous 3H-thymidine was used as the starting substrate. *D*, an amount of 3H-thymidine was observed when 3H-TMP was used as the starting substrate. All data represent the mean and SEM of three independent determinations from three individual rat tissue mitochondrial isolates. TMP, thymidine monophosphate; TTP, thymidine triphosphate.

In previous work with heart mitochondria, it was shown that 3H-thymidine was a four times better substrate for 3H-TTP synthesis than 3H-TMP, and that 3H-TMP could only serve as a substrate for TTP synthesis after conversion to thymidine (18). The present study was undertaken to determine if this finding is specific to certain tissues or widely applicable to mammalian mitochondria. The degree of thymidine and TMP conversion to TTP was studied by incubating these labeled precursors with isolated rat liver, brain, and kidney mitochondria (4 mg/ml) as described in the *Experimental procedures* section (Fig. 2). Conversion of these precursors to phosphorylated intermediates was quantitated and expressed as pmol/mg mitochondrial protein as described in the *Experimental procedures* section and compared with previously obtained heart mitochondrial data (18). As noted in the earlier studies, the amount of 3H-TDP present during these incubations was negligible and was included in the 3H-TTP values (18). In Figure 2*A*, 3H-TTP synthesis from 3H-thymidine varied significantly in mitochondria from different tissues. The conversion of 3H-thymidine to 3H-TTP in kidney mitochondria was nearly completed at 2 h and was 2.6 times (*p* < 0.001) the heart mitochondrial conversion rate, whereas the conversion rates in brain and liver at 2 h were 1.7x (*p* < 0.05) and 1.1x, NS, respectively. A similar trend in TTP synthesis was obtained when 3H-TMP was the starting substrate (Fig. 2*B*). As was noted previously for heart mitochondria, thymidine was preferred 2.5-fold over TMP for TTP synthesis in liver mitochondria after 2 h of incubation (*p* < 0.02). However, there was no difference between 3H-thymidine and 3H-TMP as substrates for 3H-TTP synthesis in brain and kidney mitochondria (*blue and black curves* in Fig. 2, *A* and *B*). As the conversion of TTP from thymidine requires the synthesis of TMP, we have measured 3H-TMP levels as a function of time in each of the tissue mitochondria. The amount of TMP present at any point in time in Figure 2*C* is a reflection of its phosphorylation from thymidine, its dephosphorylation back to thymidine, and its further phosphorylation to TDP and TTP. For liver and heart mitochondria, these data suggest that the addition of the second phosphate is slower than the addition of the first phosphate and 3H-TMP accumulates (*green and red curves* in Fig. 2*C*). However, in brain and kidney mitochondria, once TMP reaches its maximal level at 60 min, the second phosphorylation rate now equals the first (*blue and black curves* in Fig. 2*C*).

Shown in Figure 2*D* is the amount of 3H-thymidine generated from 3H-TMP. A small amount of 3H-thymidine was generated in the first hour with modest differences between liver, brain, and kidney mitochondria. 3H-thymidine levels plateaued in brain and liver mitochondria, suggesting an equilibrium between phosphorylation and dephosphorylation (*blue and green curves* in Fig. 2*D*). In kidney mitochondria, the level of 3H-thymidine decreased substantially in the second hour as the 3H-pool is nearly completely converted to 3H-TTP (*black curve* in Fig. 2*D*).

### Conversion of 3H-TMP to 3H-TTP in intact mitochondria isolated from liver, brain, and kidney in the presence and absence of azidothymidine

**Figure 3:**
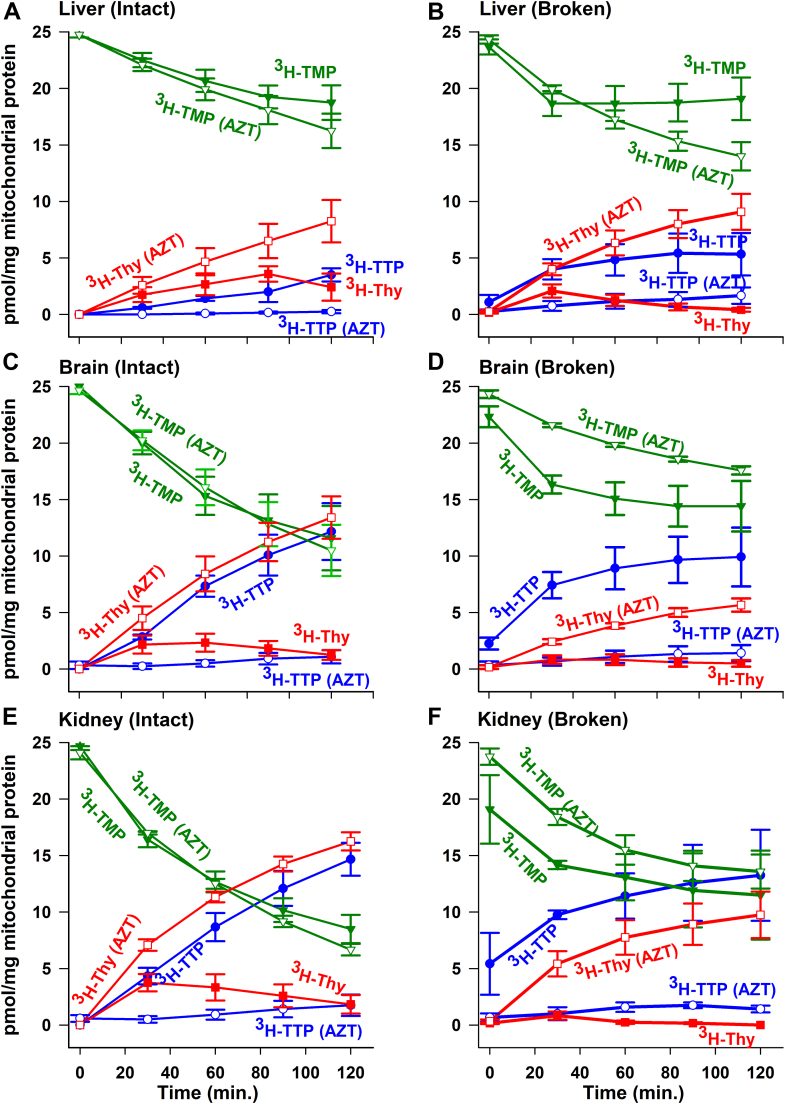
**Effect of AZT on 3H-TMP metabolism in intact and broken tissue mitochondria**. Intact rat liver, brain, and kidney mitochondria were isolated, and broken tissue mitochondria were prepared as described in the *Experimental procedures* section. Both intact and broken tissue mitochondria were incubated, processed, and products identified and quantitated as described under the *Experimental procedures* section, and results were plotted as in the legend for Figure 2. Either intact or broken tissue mitochondria were incubated with 100 nM 3H-TMP as the starting substrate for 0, 30, 60, 90, and 120 min with the presence and absence of 200 μM AZT (*A*: intact liver; *B*: broken liver; *C*: intact brain; *D*: broken brain; *E*: intact kidney; and *F*: broken kidney). AZT is a potent inhibitor of TK2; thus, 3H-thymidine that arose from 3H-TMP dephosphorylation is trapped as 3H-thymidine. All data represent the mean and SEM of three independent determinations from three individual rat tissue mitochondrial isolates. AZT, azidothymidine; TK2; thymidine kinase 2; TMP; thymidine monophosphate.

In earlier work in heart mitochondria, we used azidothymidine (AZT) to inhibit TK2 to show that the synthesis of 3H-TTP from 3H-TMP occurred solely by dephosphorylation of 3H-TMP to 3H-thymidine followed by rephosphorylation to 3H-TMP and then onto 3H-TDP and 3H-TTP (18). As demonstrated in Figure 2, *A* and *B*, thymidine was a preferred substrate for TTP synthesis in liver and heart mitochondria, whereas there was no difference between 3H-thymidine and 3H-TMP in brain and kidney mitochondria. In interpreting these data, much depends on the rate of dephosphorylation of 3H-TMP to 3H-thymidine and its subsequent rephosphorylation to 3H-TMP. The rate of dephosphorylation of 3H-TMP was measured by exogenously adding 3H-TMP to liver, brain, and kidney intact mitochondria (Fig. 3, *A* and *C*, *E*) in the presence of AZT to block thymidine rephosphorylation back to TMP. The amount of 3H-thymidine (*red open square*) observed in liver, brain, and kidney mitochondria in the presence of AZT is a reflection of the rate of 3H-TMP dephosphorylation. The rate of 3H-thymidine accumulation in the presence of AZT in the liver was quite similar to accumulation in the heart (18), whereas the accumulation of thymidine in the presence of AZT in brain and kidney mitochondria was 1.8 (*p* < 0.005) and 2.5 (*p* < 0.001) times that observed in the liver and heart, demonstrating significantly faster TMP dephosphorylation in kidney and brain mitochondria. Consistent with previous observations in heart mitochondria, the presence of AZT completely blocked 3H-TTP synthesis in all mitochondria (*blue open circle* Fig. 3, *A* and *C*, *E*), demonstrating that 3H-TMP cannot be directly phosphorylated to 3H-TTP and has to be dephosphorylated to 3H-thymidine as an intermediate. Given these results, the preference for 3H-thymidine over 3H-TMP in mitochondria is a direct reflection of the rate of TMP dephosphorylation. Thus, liver and heart mitochondria with a slower rate of TMP dephosphorylation show a decided preference for 3H-thymidine in 3H-TTP synthesis, whereas the much faster 3H-TMP dephosphorylation in brain and kidney mitochondria provides 3H-thymidine at a rate sufficient to eliminate the preference of 3H-thymidine over 3H-TMP. As a result, in brain and kidney mitochondria, 3H-TMP is converted to 3H-thymidine in the presence of AZT (*red open square* in Fig. 3, *B* and *C*) and into 3H-TTP in the absence of AZT (*blue filled circle* in Fig. 3, *B* and *C*).

### Conversion of 3H-TMP to 3H-TTP in broken mitochondria isolated from liver, brain, and kidney in the presence and absence of AZT

It was previously shown in heart mitochondria (18) and extended here to liver, brain, and kidney mitochondria as presented in Figures 2, 3, *A*, *C*, and *E* that TMP appeared to be compartmentalized such that TMP synthesized from thymidine was preferentially used for TTP synthesis. One possible hypothesis of this compartmentalization is to suggest that thymidine but not TMP is transported across the inner mitochondrial membrane. If correct, TMP would have to dephosphorylate to thymidine and the thymidine would be transported to the matrix for rephosphorylation to TMP and on to TDP and TTP. To test this hypothesis, experiments were conducted under the same conditions as the incubation of intact tissue mitochondria, except that mitochondrial membrane integrity was destroyed by freezing and thawing as described in the *Experimental procedures* section. As the loss of intactness dramatically reduces the energy charge of the system, energy charge was restored and maintained through an addition of oligomycin A, phosphocreatine, and creatine phosphokinase. This was shown in previous work in heart mitochondria to be effective in maintaining the energy charge (18) and worked identically for mitochondria in this study as well. This treatment should provide 3H-TMP free access to the enzyme systems present in the mitochondrial matrix. If the inner mitochondrial membrane was responsible for 3H-TMP compartmentalization, then synthesis of 3H-TTP from 3H-TMP should not be affected by AZT. As shown in broken mitochondria from liver (Fig. 3*B*), brain (Fig. 3*D*), and kidney (Fig. 3*F*), there was no significant difference between intact and broken mitochondria in the amount of 3H-TMP converted to 3H-TTP in the absence of AZT (*blue filled circle* in Fig. 3, *A*–*F*). However, the addition of AZT to the broken mitochondria nearly completely blocked the conversion of 3H-TMP to 3H-TTP in all three broken mitochondrial preparations (*blue open circle* in Fig. 3, *D*–*F*) just as it did in the intact preparations (*blue open circle* in Fig. 3, *A*–*C*). This indicated that even when the mitochondrial membrane was disrupted, the breakdown of 3H-TMP to 3H-thymidine was still required for 3H-TTP synthesis, implying a requirement for TK2 resynthesis of TMP in order for TTP to be made.

Much of the 3H-TTP formed from 3H-TMP in broken mitochondria in the absence of AZT (*blue filled circle* in Fig. 3, *B* and *D*, *F*) should now be observed as 3H-thymidine in the presence of AZT (*red open square* in Fig. 3, *B* and *D*, *F*) as expected for AZT inhibition of TK2. These two curves track closely in intact mitochondria (Fig. 3, *A* and *C*, *E*). However, in broken mitochondria from brain and kidney, the amount of 3H-thymidine in the presence of AZT was about half what was expected when compared with the amount of 3H-TTP formed from 3H-TMP in the absence of AZT (*p* < 0.01 for kidney and brain). This appears to be caused primarily by an increased amount of 3H-TTP appearing at the end of the freeze–thaw protocol in the absence of AZT that does not appear as 3H-thymidine in the presence of AZT. While the reason for this is not understood, it may be related to a more rapid breakdown of thymidine to thymine and other products in the broken mitochondria compared with intact mitochondria. If we correct for this difference, the amount of 3H-thymidine generated in the presence of AZT closely matches the 3H-TTP synthesized in the absence of AZT through the rest of the time course. Interestingly, in the absence of AZT, the steady-state level of 3H-thymidine produced from 3H-TMP in intact mitochondria from all three tissues was significantly higher than in broken mitochondria (liver, >2.5X, *p* < 0.02; brain, >3X, *p* < 0.002; and kidney >9X, *p* < 0.001). This suggests that a portion of the 3H-thymidine generated from 3H-TMP in intact mitochondria is produced in the extra matrix space and is unavailable for immediate reaction with matrix TK2. Alternatively, it is possible that thymidine breaks down to thymine and other products more readily in broken mitochondria.

### Two antibody proximity PCR labeling indicates an intermolecular interaction between TK2 and CMPK2 in the mitochondria

**Figure 4:**
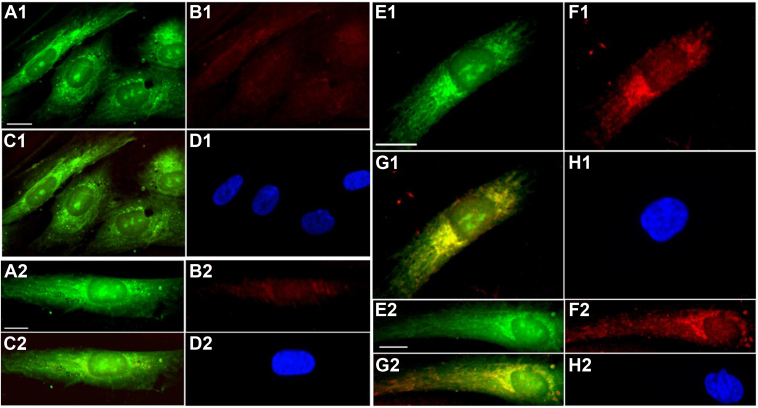
**Proximity labeling analysis of interactions between mitochondrial CMPK2 and TK2 in primary human fibroblasts**. Cells were incubated with the following probe combinations: *A*1–*D*1: negative control experiment (control 1), using a plus strand probe coupled to a CMPK2 antibody and an uncoupled minus strand probe. *A*2–*D*2: negative control experiment (control 2), using minus strand probe coupled to TK2 antibody and uncoupled plus strand probe. *E*1–*H*1 and *E*2–*H*2: cells labeled with CMPK2 antibody (*E*1–*H*1) coupled to plus strand probe and TK2 antibody coupled to minus strand probe (*E*2–*H*2). Ligation and amplification steps were performed with Duolink *In Situ* Detection Reagent *Red*. After ligation and amplification steps, cells were incubated with BioTracker 488 *Green* to detect mitochondria, DAPI stain (*blue*) to detect nuclei and viewed by fluorescence microscopy. *A*1–2 and *E*1–2: BioTracker 488 Green fluorescence (mitochondria). *B*1–2 and *F*1–2: Doulink *Red* fluorescence. *C*1–2 and *G*1–2: *Green and red signals*, *yellow* shows overlap in *G*1–2 but not *C*1–2. *D*1–2 and *H*1–2: DAPI stain of nuclei (*blue*). The bar indicates 5 μm. CMPK2, cytidine/uridine monophosphate kinase 2; DAPI, 4′,6-diamidino-2-phenylindole; TK2, thymidine kinase 2.

We employed two-antibody proximity PCR labeling to detect potential intermolecular interactions between TK2 and CMPK2. Minus or plus strand primers were covalently linked to TK2 or CMPK2 antibodies that were then incubated with fixed and detergent-permeated primary human dermal fibroblasts. After incubation, rolling-circle amplification reactions were performed with Duolink *In Situ* Detection Reagent Red to illuminate TK2 and CMPK2 antibodies bound to their respective antigens that are present in close proximity on the cells. As negative controls, cells were incubated with antibody-free primer paired with either CMPK2 (Fig. 4: A1, B1, C1, D1) or TK2 (Fig. 4: A2, B2, C2, D2) antibody coupled to the opposite primer. When incubated with uncoupled opposite primer, TK2 or CMPK2 antibodies produced a weak or background-level red signal (Fig. 4: B1, B2). In contrast, incubation with TK2 antibodies coupled to plus strand primers and CMPK2 antibody coupled to minus strand primers (Fig. 4: E1, F1, G1, H1, E2, F2, G2, H2) produced a prominent red signal (Fig. 4: F1, F2). BioTracker 488 Green was used to stain mitochondria (BioTracker 488 Green; Fig. 4: A1, A2, E1, E2), and 4′,6-diamidino-2-phenylindole was used to stain nuclei (Fig. 4: D1, D2, H1, H2). Alignment of proximal TK2 and CMPK2 signals with the mitochondrial compartment is indicated by *yellow color* (Fig. 4: G1, G2); this is not observed in the negative controls (Fig. 4: C1, C2). Nonparametric statistical analyses of the images confirmed significant alignment of mitochondrial and proximal TK2 and CMPK2 signals (Table 1). Together, the data indicate that the proximity of TK2 and CMPK2 in the mitochondria is consistent with an intermolecular interaction between the two proteins at that site.

**Table 1:** Kruskal–Wallis nonparametric analysis of random fields (N)

Analysis	Prox on mito	Control 1 on mito	Control 2 on mito
Alignment (%)	24.1	12.8	11.2
Fields observed (n)	12	6	6
Kruskal–Wallis (*P*)	0.0105		

Several fields of cells from each of the experiments shown in Figure 4 were scored for overlap between green and red signals (ImageJ). Percent alignment with mitochondrial stain on proximity-labeled cells (Prox on mito) was compared with that of control cells (control 1 and control 2, as described in the Fig. 4 legend) using Kruskal–Wallis nonparametric analysis (vassarstats.net).

### Extraction of TK2 and CMPK2 activity from fresh mitochondria

**Figure 5:**
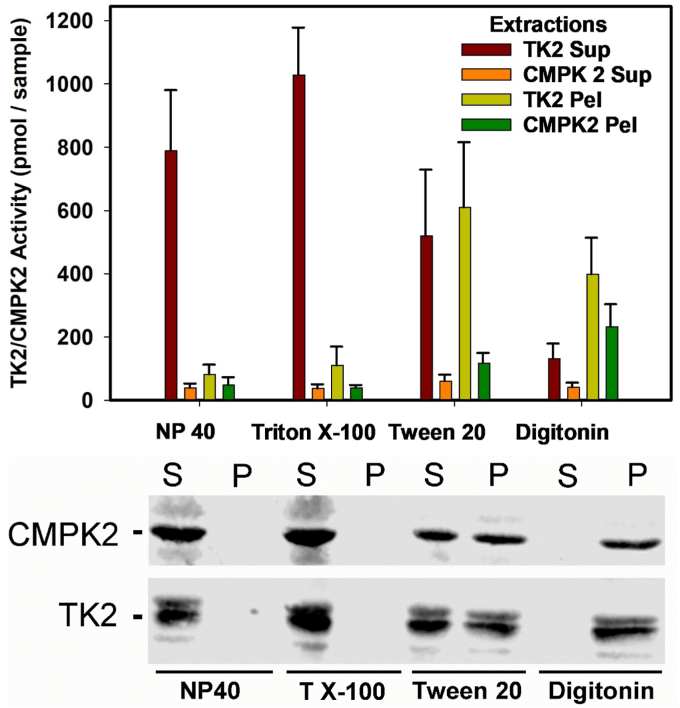
**Comparison of extraction of TK2 and CMPK2 activity from isolated mitochondria with Western blot**. *Top*, aliquots of rat liver mitochondria were extracted with four different detergents and separated into supernatants and pellets as described in the *Experimental procedures* section. TK2 activity was estimated by following the conversion of 3H-thymidine or 3H-dC to phosphorylated products and is shown as TK2 activity in the figure. CMPK2 activity in the figure was estimated by the conversion of 3H-TMP to 3H-TDP + TTP and 3H-dCMP to 3H-dCDP + dCTP. *Bottom*, Western blot analysis of extracted liver mitochondria: Aliquots of the supernatants (S) and pellets (P) shown in the *top* were analyzed by Western blots using antibodies to TK2 and CMPK2. Samples were loaded to represent equivalent amounts of starting material as described in the *Experimental procedures* section. Images of the uncropped Western blots with migration markers are included in Supporting Information S1. CMPK2, cytidine/uridine monophosphate kinase 2; DAPI, 4′,6-diamidino-2-phenylindole; TK2, thymidine kinase 2; TMP, thymidine monophosphate.

To determine if TK2 and CMPK2 enzyme activity and protein could be extracted from mitochondria, we subjected fresh liver mitochondria to four different detergent extractions: NP-40, Triton X-100, digitonin, and Tween-20, and separated them into supernatant and pellet as described in the *Experimental procedures* section. An aliquot of each was assayed for enzyme activity as described above for intact or broken mitochondria (Fig. 5, *top*). A second aliquot was used for Western blot analysis (Fig. 5, *bottom*, whole blot in supporting information). As shown by the Western blot in Figure 5, bottom, the strong detergents, NP-40 and Triton X-100, completely extracted both TK2 and CMPK2 proteins into the supernatant fraction. Tween-20 yielded a partial extraction, with proteins in both fractions, whereas digitonin failed to extract either protein into the supernatant. As expected, the enzyme activity of TK2 followed the extraction as noted on the Western blot, with the bulk of the TK2 activity in the NP-40 and Triton X-100 supernatant and in the digitonin pellet, while it was split between supernatant and pellet in the Tween-20 extraction (Fig. 5, top). Interestingly, while the CMPK2 protein distributed the same as TK2, there was very little enzyme activity, if any, in any of the supernatants. Enzyme activity of CMPK2 was observed mostly in the digitonin and Tween-20 pellets (Fig. 5, *top*). One interpretation of these data is that the extraction disrupted the interaction between TK2 and CMPK2, resulting in loss of CMPK2 activity. Alternatively, even though an energy-generating system was supplied, it was noted that energy charge was harder to maintain in the supernatants, which could contribute to low activity of CMPK2, although it did not appear to affect TK2 activity.

## Discussion

The results of this work demonstrate that the synthesis of TTP from thymidine in mitochondria isolated from tissues that are mostly postmitotic requires an unusual compartmentation of the intermediate TMP. This corroborates our results first shown in isolated heart mitochondria in which a large bolus of exogenous unlabeled TMP had no effect on the conversion of 3H-thymidine to 3H-TTP (17), indicating that the exogenous TMP and the 3H-TMP synthesized from 3H-thymidine did not mix. In subsequent work in isolated heart mitochondria, it was shown that 3H-thymidine was a much better substrate for 3H-TTP synthesis than the downstream intermediate 3H-TMP, and when AZT was added to inhibit TK2, the conversion of exogenous 3H-TMP to 3H-TTP was completely blocked with the accumulation of 3H-thymidine. The studies here extend these findings to three additional tissues: mitochondria, liver, brain, and kidney. While the preference for 3H-thymidine over 3H-TMP for TTP synthesis was demonstrated in these studies for isolated liver mitochondria (Fig. 2, panels A and B), there was no preference for 3H-thymidine over 3H-TMP in isolated mitochondria from the brain and kidney. However, this was related to the much faster dephosphorylation of 3H-TMP in isolated brain and kidney mitochondria, observed in the presence of AZT to prevent the rephosphorylation of 3H-thymidine back to TMP, as shown in Figure 3, C (brain) and E (kidney). Thus, the dephosphorylation of 3H-TMP provided sufficient 3H-thymidine in mitochondria from brain and kidney that the forward reaction through TK2 was not limited and proceeded to the same extent as observed with an exogenous addition of 3H-thymidine. Higher levels of 5′ nucleotidase activity have also been shown in the brain and kidney in the mouse (23).

An attractive reason to account for the apparent compartmentation of 3H-TMP in this synthetic pathway would be the inability to transport TMP across the inner membrane, such that exogenous TMP would not have access to the matrix enzymes and thus would have to dephosphorylate to thymidine to enter the matrix. However, others have reported that TMP is transported into the matrix (8) making this less likely. To confirm this finding, experiments were conducted on frozen and thawed liver, kidney, and brain mitochondria that lacked membrane intactness. In these studies, 3H-TTP was synthesized from both 3H-thymidine (data not shown) and 3H-TMP (Fig. 3, *B*, *D*, and *F*). However, the addition of AZT to these broken mitochondria, to inhibit TK2, nearly completely blocked synthesis of 3H-TTP, and label accumulated as 3H-thymidine (Fig. 3, *B*, *D*, and *F*). These results indicate that the compartmentation of TMP is not mediated by the failure of inner membrane transport.

**Figure 6:**
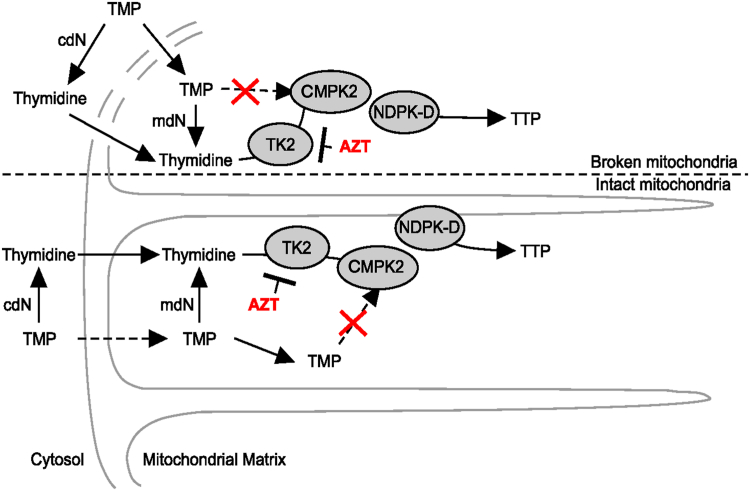
**Proposed model of the interaction of TK2 and CMPK2 in thymidine and TMP salvage in rat mitochondria**. Shown is the proposed model of TK2 and CMPK2 interaction to account for TMP compartmentalization in intact and broken rat tissue mitochondria. cdN, cytosolic deoxynucleotidase; CMPK2, cytidine/uridine monophosphate kinase 2; ENT1/2, equilibrative nucleoside transporter 1/2; mdN, mitochondrial deoxynucleotidase; NDPK-A, B, cytosolic nucleoside diphosphate kinase; NDPK-D, mitochondrial nucleoside diphosphate kinase; TK1/2, thymidine kinase 1/2; TMP, thymidine monophosphate; TMPK, cytosolic thymidylate kinase; TMPK2, presumptive mitochondrial thymidylate kinase (CMPK2).

Using proximity labeling and immunofluorescence microscopy analysis, we have shown that TK2 and CMPK2 interact in the mitochondria (Fig. 4, Table 1). This experiment required mitotic cells in culture for visualization. As TK2 and CMPK2 are also expressed in mitotic cells, we reasoned that the interaction between them should be the same in mitotic cells as in nonmitotic cells. In addition, extraction of mitochondria with strong detergents completely extracted both TK2 and CMPK2 into the supernatant, but only TK2 remained active as an enzyme, again suggesting an interaction between TK2 and CMPK2 that is disrupted by the detergent extraction. From these data, we have proposed an alternative hypothesis illustrated in Figure 6. This follows from the work by Chen *et al*. (11), which identified and isolated a mitochondrial enzyme they named TMPK2, as its sequence placed it in the family of TMP kinases. They isolated a recombinant protein and demonstrated that when overexpressed, it increased TTP synthesis twofold in both cells and isolated mitochondria, suggesting that the enzyme functioned as a TMPK; however, they could not show that the purified recombinant protein had enzymatic activity with exogenous TMP. It was subsequently shown by Xu *et al*. (12) that the sequence identified by Chen *et al*. (11) was already known as CMPK2, known to phosphorylate dUMP and dCMP, but like Chen *et al*., not TMP. Frisk *et al*. (24, 25, 26) noted that humans with loss-of-function mutations in the known cell cycle–regulated TMPK had microcephaly and hypotonia but survived with other tissues unaffected (24, 25). They proposed that there must be an additional enzyme with TMPK activity but were unable to identify it. We propose (Fig. 6) that CMPK2 is that enzyme, which associates with TK2 and acquires TMPK activity and phosphorylates newly made enzyme-bound TMP to TDP. In this manner, thymidine would bind to the TK2–CMPK2 enzyme complex and would not dissociate until it was converted to TDP. This may be related to the demonstration by us (17, 27) and others (28) that TK2 displays negative cooperativity with regard to thymidine as a substrate. As unbound CMPK2 does not have TMPK activity, exogenous TMP would not serve as a substrate and would account for the observed TMP compartmentalization. If this hypothesis is correct, it would allow the pathway to function more efficiently, as the TMP synthesized by TK2 would not have the opportunity to be dephosphorylated by mitochondrial 5′ nucleotidase back to thymidine, decreasing a potential futile cycle. In this regard, the role of mitochondrial 5′ nucleotidase may be to ensure that all TTPs synthesized are mediated *via* TK2 by dephosphorylating exogenous TMP to thymidine. While these data support the hypothesis presented, future work with reconstitution experiments using purified CPMK2 and TK2 is needed to test the hypothesis presented in Figure 6. It is interesting to speculate that the mitochondrial thymidine to TTP salvage pathway might exist as a metabolon akin to the glucosome (29), purinosome (30), or the heme biosynthetic pathway (31). The final enzyme in this pathway is the mitochondrial nucleoside diphosphate kinase D complex (NME4). While the association of this enzyme with TK2 or CMPK2 has not been investigated, this enzyme is known to form a complex that is associated with succinyl-CoA synthetases, SUCLG1, SUCLG2, and SUCLA2, as well as γ-aminobutyric acid transaminase (13). Interestingly, expression of CMPK2 has been reported to mediate immunomodulatory and antiviral activities through interferon-dependent and independent pathways (32). CMPK2 has also been shown to accelerate liver ischemic/perfusion injury by activating an NLRP3 (nucleotide-binding domain, leucine-rich–containing family, pyrin domain–containing-3 protein) signaling pathway (33). Activation of the NLRP3 pathway requires new mitochondrial protein synthesis, presumably mediated in part by expression of CMPK2 (34).

The results described in this study were observed in mitochondria from four different tissues: heart, liver, brain, and kidney. The extent to which this applies to other cells and tissue mitochondria remains unclear. However, in tissues and cells that actively replicate nuclear DNA and have much higher deoxynucleoside triphosphate pools, TTP can be synthesized from sources other than thymidine. Work from others has shown that the mitochondrial TTP pool in replicating cells comes mostly from the cytosolic synthetic pathways (7, 35).

The results of this work have important ramifications for humans suffering from TK2 deficiency, a mitochondrial DNA depletion disease, as it suggests that the improvement observed in affected patients with deoxynucleoside treatment in nonmitotic tissues is not mediated by the mitochondrial salvage pathway and is perhaps related to residual phosphorylation activity of cytosolic deoxynucleoside kinases, such as TK1 and dCK.

## Experimental procedures

### Chemicals and biochemicals

3H-thymidine and 3H-TMP were purchased from Moravek Biochemicals. Zidovudine (AZT) was purchased from Synthonix. All unlabeled chemicals used in this study were purchased from Sigma–Aldrich.

### Intact tissue mitochondria

In-house outbred Sprague–Dawley rats were raised in the institution vivarium, and experiments were carried out in accordance with an Institutional Animal Care and Usage Committee–approved animal protocol. Coupled tissue mitochondria were isolated from adult female rat tissues (liver (36), kidney (18), and brain (37)) using differential centrifugation methods described previously, except nagarse was not used for liver mitochondria. The intactness of isolated tissue mitochondria was confirmed by measuring the respiratory control ratio using glutamate and malate as substrates as previously described (18) in a high-resolution respirometer (Oxygraph-2K; Oroboros). Tissue mitochondria used for these experiments all have a respiratory control ratio value of six and above. Mitochondrial protein concentration was determined by Lowry assay using bovine serum albumin as a standard.

### Incubation of isolated intact and broken mitochondria

Intact rat tissue mitochondria were incubated at a final concentration of 4 mg mitochondrial protein/ml in incubation media described previously (18) with an addition of 100 nM of either 3H-thymidine or 3H-TMP (∼2200 dpm/pmol). Where indicated in the results and figure legends, 200 μM AZT was added to the incubation to block TK2 activity. The incubation was terminated at specific times by removing an incubation aliquot with the addition of an equal volume of 10% trichloroacetic acid to lyse the mitochondria and precipitate insoluble macromolecules. Trichloroacetic acid–treated samples were centrifuged to remove the precipitates, and the resultant supernatant was neutralized with ion-exchange resin (AG-11A8; Bio-Rad) and filtered through a 0.2 μm nylon syringe filter. The amount of radioactivity in each sample was determined by counting an aliquot in scintillation fluid (Insta-Gel Plus; PerkinElmer) on a liquid scintillation analyzer (Beckman Coulter 6500).

### Preparation of broken mitochondria

Rat liver, brain, and kidney mitochondria were isolated, quantified, and incubated as described previously with the exception that prior to freezing and thawing, oligomycin A (50 μM), phosphocreatine (12 mM), and creatine phosphokinase (50 units/ml) were added to the incubation mixture to maintain mitochondrial energy charge. Broken mitochondria were obtained by freezing and thawing three times in liquid nitrogen as described previously (18). Aliquots were removed in the same manner as for intact tissue mitochondria.

### UPLC deoxynucleotide analysis and radioactivity quantification

Quantitation of 3H-thymidine, 3H-TMP, and its intermediates and phosphorylated products was analyzed as described previously (18) Briefly, this was accomplished by UPLC (1290 Infinity; Agilent) equipped with a C18 reverse-phase column (ZORBAX Eclipse Plus, 3.0 × 150 mm, 1.8 μm; Agilent) coupled to an inline diode array and liquid scintillation counter (β-RAM5, LabLogic). The mobile phases were composed of 5 mM tetrabutylammonium acetate, 60 mM ammonium acetate, pH 5.0 (A), and 5 mM tetrabutylammonium acetate in methanol (B) using a gradient program. The flow rate and column temperature were set at 0.5 ml/min and 30 °C, respectively. Diode array was used to quantitate levels of ADP and ATP, and the 3H-signals were detected by β-RAM and quantified using Laura software (LabLogic).

### Preparation of antibodies for proximity labeling

Rabbit TK2 antibody was purchased from Novus Biologicals (NBP1-92505), and rabbit CMPK2 antibody was purchased from MyBiosource (MBS153401). Antibodies were validated by the commercial providers with references to their use in other publications. Minus or plus strand primers were ligated to the TK2 or CMPK2 antibodies using Duolink *In Situ* Probemaker reagents as described by the manufacturer (DUO92010 and DUO92009; MilliporeSigma). Plus and minus primer-coupled antibodies were purified from free probe using Ultracel-10 membrane centrifugal filters (MRCPRT010; MilliporeSigma).

### Proximity immunofluorescence analysis

Cultured primary human fibroblasts were acquired from the American Type Culture Collection (PCS-201-012) and cultured in Dulbecco's modified Eagle's medium supplemented with 10% fetal bovine serum. Cells were cultured on 1 cm round cover slips in 24-well plates, fixed for 20 min with freshly made 3.7% paraformaldehyde in PBS (at room temperature), and treated with 0.05% Triton X-100 in PBS for no more than 3 min at room temperature. The parameters of the latter step are important for the mitochondrial staining. Cover slips were washed twice with PBS, then incubated with Tris-buffered saline (20 mM Tris, 150 mM saline, pH 7.4) containing 5% acetylated bovine serum albumin (MilliporeSigma) overnight at 4 ^o^C. Cells were washed with Tris-buffered saline twice, then incubated with Duolink Blocking solution (MilliporeSigma) for 60 min at 37 ^o^C. Cover slips were washed and incubated overnight with CMPK2 and TK2 plus or minus strand antibodies in Duolink antibody diluent. Slides were washed, and ligation and amplification of the probes were performed using the Duolink Red *In Situ* Detection Reagents and the procedures recommended by the manufacturer (MilliporeSigma). Mitochondria were stained with BioTracker 488 Green Mitochondrial Dye (MilliporeSigma). Slides were mounted and viewed with a Leitz DM5000B series fluorescence microscope.

### Extraction of TK2 and CMPK2 activity from fresh mitochondria

Four tubes of 10 mg of liver mitochondrial protein were centrifuged at 10,000*g* for 5 min. The pellets were resuspended in malignant serous effusion (220 mM mannitol, 70 mM sucrose, 5 mM Mops, 2 mM EGTA, pH 7.2) containing Pierce protease inhibitor cocktail minus EDTA (AEBSF [4-(2-aminoethyl)-benzenesulfonyl fluoride hydrochloride], aprotinin, bestatin, E-64, leupeptin, and pepstatin A) plus one of four detergents: 0.5% NP-40, 0.1% Triton X-100, 0.25% Tween-20, and 0.04% digitonin. The pellets were dispersed by 10 strokes with a mini glass dounce. Following an incubation for 1 h on ice, the extracted mitochondria were pelleted by centrifugation at 10,000*g* for 5 min, and the supernatant was transferred to a clean tube. The pellets were resuspended in 0.5 ml of malignant serous effusion. A 40 μl aliquot of each supernatant and pellet fraction was incubated for 1 h with either 3H-thymidine, 3H-dC, 3H-TMP, or 3H-dCMP, exactly as described for intact mitochondria. The 3H deoxynucleotides were extracted as described for intact mitochondria and analyzed by UPLC. TK2 activity was estimated by following the conversion of 3H-thymidine or 3H-dC to phosphorylated products and is shown as TK2 activity in Figure 5, *top*. CMPK2 activity in Figure 5, *top* was estimated by the conversion of 3H-TMP to 3H-TDP + TTP and 3H-dCMP to 3H-dCDP + dCTP.

### Western blot analysis of extracted liver mitochondria

Pellets were suspended in 1X Laemmli buffer, and 4X Laemmli was added to the supernatants. The final volumes of pellets and supernatants were adjusted to equally represent the amount of starting material from which they were derived. Loaded samples were separated by SDS-PAGE and transferred electrophoretically to nitrocellulose membranes. The blots were probed with the TK2 or CMPK2 antibodies described previously. After incubation with primary antibodies, the blots were washed and incubated with fluorescent secondary antibodies purchased from Licor. Blots were imaged and quantified using the Odyssey XF System (Licor).

### Data analysis of deoxynucleoside/deoxynucleotide quantitation

The amount of product observed was expressed as dpm/mg mitochondrial protein and converted to pmol/mg mitochondrial protein based on 3H-precursor specific radioactivity as described previously (18). The commercial stock of 3H-thymidine used for these experiments had a substantial early eluting breakdown product that reduced the concentration of 3H-thymidine. The breakdown peak was not affected by incubation with mitochondria, indicating that 3H-thymidine was not broken down in isolated mitochondria. As the rate of thymidine phosphorylation is linear with 3H-thymidine concentration in this range (17, 36, 37), we corrected the 3H-thymidine concentration to match the 100 nM 3H-TMP concentration for comparison. Data presented in the figures represent the mean and SEM of at least three independent determinations from three individual rat tissue mitochondrial isolates. Significant differences in results were computed *via* a Student’s *t* test.

## Data availability

All data are contained within the article.

## Supporting information

This article contains supporting information.

## Conflict of interest

The authors declare that they have no conflicts of interest with the contents of this article.
