# Supplementary material for: Compartmentalized thymidine phosphorylation by mitochondrial nucleotide kinases TK2 and CMPK2
Source: J Biol Chem. 2025 Sep 16;301(11):110733. doi: 10.1016/j.jbc.2025.110733 (PMC12547304; doi:10.1016/j.jbc.2025.110733)
Supplement: Supporting Information [file mmc1.pdf]

Supporting Information for:

Interaction of mitochondrial TK2 and CMPK2 and compartmentalization of TMP in the synthesis of TTP in rat mitochondria. Avery S Ward, Vasudeva G Kamath, Chia-Heng Hsiung, Zachary J. Lizenby, Alexander G Gillish, D. Stave Kohtz, and Edward E McKee

One Figure

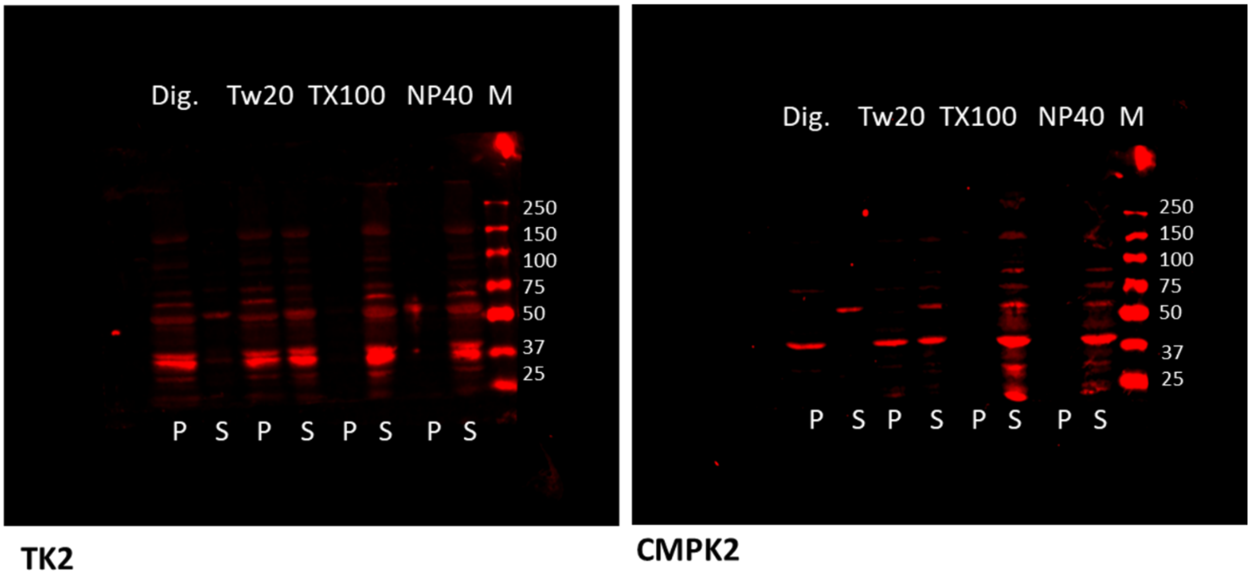

S1. **Comparison of Extraction of TK2 and CMPK2 activity from isolated mitochondria with Western Blot.** Complete Western Blot of the abstracted blot shown as Figure 5 in the manuscript. Details for the blot are described in the Experimental Procedures and the Legend to Figure 5.
